# Advancing the application, quality and harmonization of implementation science measures

**DOI:** 10.1186/1748-5908-7-119

**Published:** 2012-12-11

**Authors:** Borsika A Rabin, Peyton Purcell, Sana Naveed, Richard P Moser, Michelle D Henton, Enola K Proctor, Ross C Brownson, Russell E Glasgow

**Affiliations:** 1 CRN Cancer Communication Research Center, Institute for Health Research, Kaiser Permanente Colorado, Legacy Highlands Building, 10065 E. Harvard Ave., Suite 300, Denver, Colorado, USA; 2 Division of Cancer Control and Population Sciences, National Cancer Institute, Rockville, MD6130 Executive Blvd, Bethesda, MD, 20892, USA; 3 George Warren Brown School of Social Work, Washington University in St. Louis, Campus Box 1196, One Brookings Drive, St. Louis, MO, 63130, USA; 4 Prevention Research Center in St Louis, Brown School, Washington University in St. Louis, 660 S Euclid, Campus Box 8109, St Louis, MO, 63110, USA; 5 Division of Public Health Sciences and Alvin J. Siteman Cancer Center, Washington University School of Medicine, Washington University in St. Louis, Washington, USA

**Keywords:** Implementation, Dissemination, Measures, Constructs, Quality of measurement, Harmonization, Technology-mediated social participation

## Abstract

**Background:**

The field of implementation science (IS) encompasses a broad range of constructs and uses measures from a variety of disciplines. However, there has been little standardization of measures or agreement on definitions of constructs across different studies, fields, authors, or research groups.

**Methods:**

We describe a collaborative, web-based activity using the United States National Cancer Institute’s (NCI) Grid-Enabled Measures (GEM) portal that uses a wiki platform to focus discussion and engage the research community to enhance the quality and harmonization of measures for IS health-related research and practice. We present the history, process, and preliminary data from the GEM Dissemination & Implementation (D&I) Campaign on IS measurement.

**Results:**

The GEM D&I Campaign has been ongoing for eight weeks as of this writing, and has used a combination of expert opinion and crowd-sourcing approaches. To date it has listed definitions for 45 constructs and summarized information on 120 measures. Usage of the website peaked at a rate of 124 views from 89 visitors on week seven. Users from seven countries have contributed measures and/or constructs, shared experience in using different measures, contributed comments, and identified research gaps and needs.

**Conclusion:**

Thus far, this campaign has provided information about different IS measures, their associated characteristics, and comments. The next step is to rate these measures for quality and practicality. This resource and ongoing activity have potential to advance the quality and harmonization of IS measures and constructs, and we invite readers to contribute to the process.

## Background

Although individual investigators have been conducting what is now called implementation science (IS) for many years [1], the identification and convergence into a recognized field of inquiry has been relatively recent [2,3]. As is common in new areas of science, researchers have developed different IS frameworks, approaches, definitions, and measures [4-7]. In the last few years, the IS field has moved toward increasing consensus on key principles and definitions of central constructs [5,8-10]. At present, however, there is little consensus concerning the best measures of these constructs.

A commonly cited public health adage is ‘what gets measured, gets done [11].’ There are multiple reasons to encourage a focus on advancing measurement and harmonization of measures [12]. First, measurement can help refine the meaning and understanding of constructs and help enhance conceptual frameworks, models, and theories [13,14]. Second, use of reliable and valid measures can help increase precision and efficiency of studies. Possibly most importantly, harmonization of measures helps advance science by increasing comparability of findings across studies and facilitating meta-analysis, systematic reviews, aggregation of data, and data sharing [12].

A focus on measurement and harmonization of health-related measures in IS is timely given the current status of the field. For some constructs, such as ‘reach’ and ‘implementation climate,’ there are divergent or conflicting definitions and several measures [5,15]. Other constructs do not have easily accessible measures, or the existing ones apply only to a certain setting, or do not have established psychometric properties. In many IS health-related research areas, there currently is little harmonization—making comparisons and cross-study conclusions difficult or impossible. Finally, an important concern for IS and other areas of applied research is that there is little or no information that has been summarized to date about practical, actionable measures that are appropriate for use in real-world settings that have many competing demands, and in which use of ‘gold standard measures’ is not possible [16]. By definition, ‘gold standard’ measures have strong psychometrics properties most often due to their inclusion of many items, permitting more refined and comprehensive assessment of characteristics. Such measures are important for scientific understanding and, when one has sufficient time and supporting research staff to administer, enhance quality control, minimize missing data, and interpret results. It is not feasible, however, to use these types of measures in many low-resource settings and with underserved populations under typical conditions. It is therefore important to also identify ‘practical’ measures that are feasible to use in most settings and when one is measuring a large number of factors and response burden is of concern.

The purposes of this article are to: provide a rationale for efforts to enhance the quality and harmonization of measures for health-related IS; describe the Grid-Enabled Measures portal (GEM) and the GEM Dissemination and Implementation (D&I) Campaign to achieve these aims; present preliminary results and current contents of the GEM D&I measures repository; invite both researchers and practitioners to contribute to this collaborative endeavor; and conclude with implications and directions for future research, policy, and practice. While the primary focus of this project is on IS measures for health-related (including mental health), constructs and measures from other disciplines might also inform our thinking and might be included in the repository.

### The GEM portal and the use of crowd sourcing/wiki approach to science

**Figure 1:**
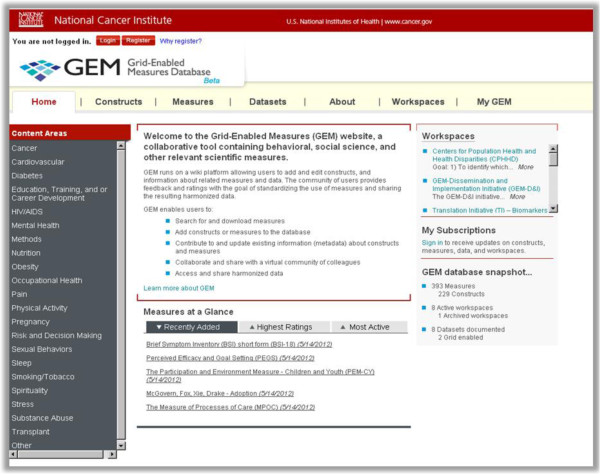
Grid-enabled Measures Database Home page screenshot.

The GEM portal was created to support behavioral and social scientists and other stakeholders as they advance their research and clinical practice through the use of standardized measures based on theoretically-based constructs (Figure 1). In this case, ‘standardized’ refers to the use of the same measure across independent protocols. GEM leverages the principles of technology-mediated social participation (TMSP), such as open access, collective intelligence, and data-driven decision making, to build a knowledge base that encourages and supports collaboration [17]. The community-generated content on GEM consists of constructs and their associated measures, along with information about (*i.e.*, meta-data) constructs and measures, such as theoretical foundation, reliability, and mode of administration, which provide the information needed—together with the qualitative data from user comments—to rate and assess each construct and measure [12]. This type of functionality is becoming ubiquitous with many websites that seek a bi-lateral exchange of information between the website and the user. Like other websites that use TMSP, GEM runs on a wiki-platform so registered users can add and edit information, including adding and editing information about new constructs, affiliated measures, and their definitions and classifying measures under constructs. GEM also provides a platform for participation to elicit input from users through the use of commenting and rating.

**Figure 2:**
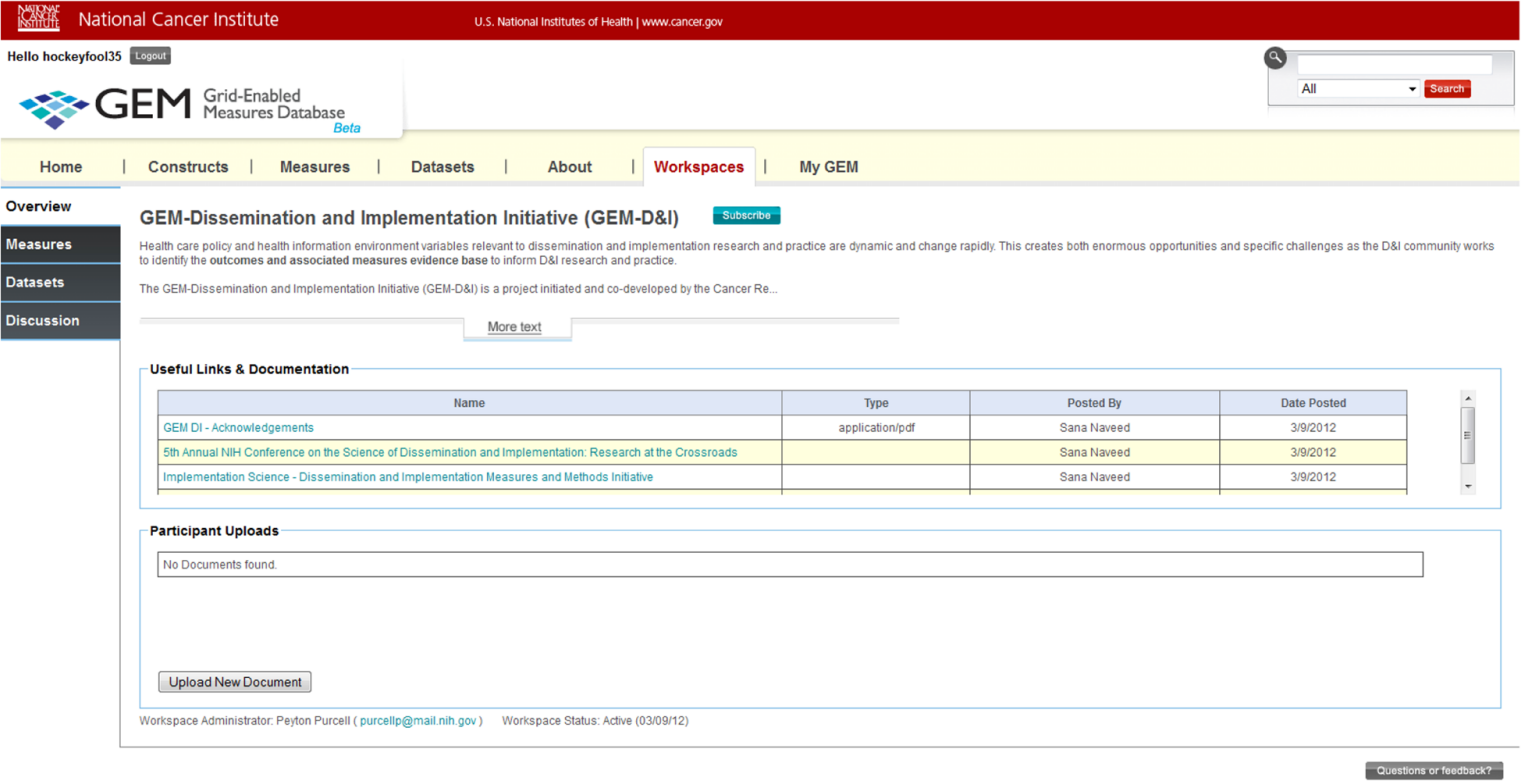
Grid-enabled Measures Dissemination and Implementation Workspace Home page screenshot.

The Dissemination and Implementation Campaign used the Workspaces in GEM, which are one of the newer features of the site (see Figure 2). Workspaces are collaborative web spaces linked to the main GEM website where researchers, clinicians, and other stakeholders can come together in an organized way to collaborate and evaluate constructs and measures that are important to their communities of practice. Through a four-step campaign process, leaders with expertise in this research area, termed ‘Champions,’ volunteer to take the lead on a number of activities: to educate the community about how to use GEM to share knowledge and build a dialogue around constructs and measures; to engage the wider scientific community to populate the database with meta-data regarding measures and constructs, and upload the actual measures (with permission or when in the public domain) for others to see and evaluate; to rate measures by assessing and evaluating the measures using a five-point rating system, and also supplying qualitative feedback through comments and a discussion board available in each Workspace; and to celebrate at a face-to-face meeting to share and discuss results. Workspaces have become a popular place to build a community of practice within specific areas of research, drive consensus toward the standardization of measures and constructs, and facilitate data sharing. GEM currently has eight active workspaces.

This participatory or crowd-sourced approach to identifying best measures takes advantage of the collective wisdom of those who have the expertise and, under the right circumstances, this method has been shown to create more accurate outcomes [18] than traditional approaches (*e.g.*, small expert groups) to gathering feedback from stakeholders. For example, having a broad range of stakeholders whose opinions are not influenced by others (*i.e.*, independence) with a mutual goal and a way to aggregate responses has been shown to lead to favorable (*i.e.*, more accurate) outcomes [19], which in this case, translates to vetting and using the best measures. For GEM, this use of collective intelligence is designed to encourage steps toward possible measure standardization. Though standardization may have different meanings, in the case of GEM, the goal is an *a priori* agreement, and use of the exact same measure before data are collected. Through the process of sharing and rating/commenting on, measures can be used to initiate a dialogue around building consensus on select key measures. This, in turn, facilitates data that contain the same measures to be shared and analyzed in innovative ways by comparing across studies, or merging datasets to conduct innovative and more robust analyses that can advance our science. In the future, it is hoped that GEM can support researchers in answering important public health research questions that cannot be answered without such a collaborative platform. For example, by merging independent data, researchers can gain larger sample sizes (*i.e.*, increased statistical power)—something that would be particularly useful when studying hard-to-reach populations—or make comparisons across samples to help create a cumulative knowledge base. There are current efforts underway to allow GEM users to search for and share data across platforms to support these types of integrative analyses.

## Methods

To initiate the GEM D&I Campaign, a small group of IS leaders from the Kaiser Permanente Colorado Cancer Communication Research Center, the National Cancer Institute, and Washington University in St. Louis, initially added a number of constructs (n = 17) and affiliated measures (n = 63) to pre-populate the GEM D&I workspace prior to the public launch. These constructs and measures were identified using expert input and a recently published paper by Proctor *et al.* that identified critical constructs for health-related IS outcomes [20], followed by a focused, non-systematic search of the literature for additional measures using snowball sampling. This search involved the identification of additional relevant publications, constructs, and measures using search of reference lists and online resources. Definitions for initial uploaded constructs were entered by members of the research team using descriptions from referenced publications. The research team relied on the literature and their own expertise in IS to assign measures to different constructs.

With the D&I workspace pre-populated with this initial list of constructs and measures added, the GEM D&I Campaign launched in March 2012 to coincide with the 5th Annual National Institutes of Health (NIH) Conference on Science of Dissemination and Implementation (http://conferences.thehillgroup.com/obssr/di2012/about.html). At the conference exhibit booth, participants were encouraged to sign up to receive additional information via email in the weeks following the conference and become an active contributor to GEM-D&I. Over 120 conference participants signed up to receive information at the exhibit booth or through promotional emails following the conference, and 77 indicated a willingness to serve as a Champion. Those who signed up represented different regions across the country and countries around the world, and organizational affiliation ranged from large government agencies to private sector organizations, to small non-profit organizations. The largest number of interested users (61%) came from academic or research institutes, which is not surprising because this is representative of the conference audience.

**Figure 3:**
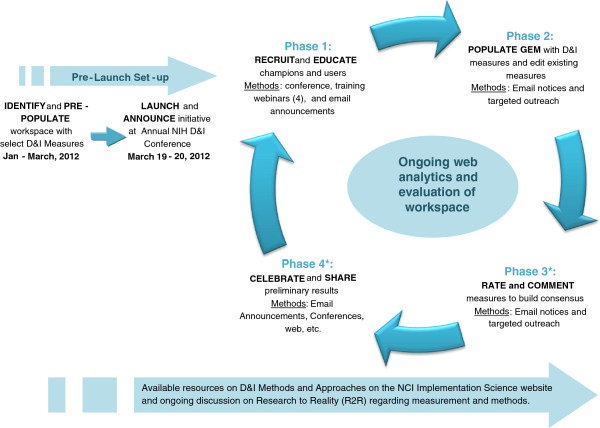
The Grid-Enabled Measures Dissemination and Implementation Campaign.

As indicated earlier, GEM campaigns are traditionally divided into four phases: educate; populate; rate; and celebrate. For the D&I Campaign, although dates are used to define each phase, the campaign is designed to be ongoing, and the workspace will continually be evaluated and assessed throughout its lifespan as the community iteratively continues to identify, refine, add and rate measures related to IS (Figure 3).

Phase 1 was initially run from the launch in mid-March through 6 April 2012. The focus of this phase was to inform and educate potential users on the campaign’s purpose and objectives, provide an overview of GEM and how it will be utilized, and opportunities and instructions for participation. This was accomplished through a webinar training offered four times over the course of one week and conducted by NCI and Kaiser Permanente staff. Targeted emails to conference participants, as well as other colleagues and outlets, were used to promote the webinars, along with attached factsheets and information regarding the D&I measures campaign. A total of 60 users registered to participate in a webinar and 52 attended a session. Presentation materials and instructions were distributed to all interested users that were unable to attend the webinars. Following the webinars, email announcements were sent with a campaign toolkit (email invitation; instruction guide (Additional file 1); factsheets (Additional file 2); GEM template slides to invite others to participate, and webinar links).

This marked the transition into phase 2, the populate phase. These announcements were distributed to the interested conference attendees, webinar participants, and additional national and international leaders in the field of D&I, who were identified by the research team as potential Champions. These Champions and interested users were tasked to help promote the D&I Campaign through GEM and the importance of harmonizing IS measures through the distribution of materials and announcements to their colleagues and networks.

The focus of this second phase, which ran from 6 April 2012 through 14 May 2012, was to increase the number of measures and constructs, and complete information (*i.e.*, meta-data) fields (*e.g.*, reliability, validity, description.) in the D&I workspace by users as well as continued work by the program team. Weekly email notices were sent to those individuals who signed-up at the D&I Conference or participated in one of the webinars with updates regarding the measures added/edited and instructions on how to add/edit their preferred D&I measures in the system. In weeks 3 and 4 of the populate phase, the notices focused on encouraging users to upload measure instruments as well as add new measures to constructs not yet populated (*e.g.*, Evidence Based Practice Attitude scale, and the constructs of Feasibility and Acceptability). Incentives for participation—beyond the possibility to contribute one’s opinion and feedback for a larger purpose—included the opportunity to be listed as a GEM D&I contributor on the NCI Division of Cancer Control and Population Sciences (DCCPS) Implementation Science website (http://cancercontrol.cancer.gov/IS/), as well as acknowledgement at an upcoming D&I conference. No other incentives were provided, and IRB approval was not required because no individually identifiable data were collected.

Phase 3, the rate phase, includes the publication of this article with a focus on continuing the dialogue around measure consensus within the IS community by allowing users to rate and comment on measures added to GEM D&I. Users are encouraged to enter both quantitative ratings and qualitative comments at any time during the campaign if they are familiar with the measure. However, the promotional push for this phase of the D&I campaign did not start until the end of the populate phase to allow for an increased number of measures and complete measure information to be uploaded before sending a wide call for ratings. We recognize that there will always be new measures identified and measures that did not get added to GEM initially, as such, this second populate phase is iterative and ongoing and individuals are encouraged to continue to add measures.

Prior to beginning the promotional efforts for the rate phase, leaders from key topic and construct areas were asked to review the measures to determine if any key IS health measures or information fields were missing that would prevent effective rating. Where gaps were identified, the D&I campaign team attempted to add additional information through further literature reviews, with an emphasis on psychometric characteristics such as validity and reliability.

During phase 3, readers are invited to rate and comment on measures. To develop rating guidelines, an open, collaborative dialogue was initiated between the core GEM D&I campaign team and lead Champions from Washington University. These discussions shaped and led to the final proposed rating guidelines wherein users are asked to provide two ratings on two separate five-point scales in regards to this measure being a 'Gold Standard Measure (1 = weak; 5 = strong) and a 'Practical Measure’ (1 = low practicality; 5 = high practicality). Table 1 includes a listing of the criteria to consider when assigning these two ratings. The first rating, 'Gold Standard,’ is based on traditional measurement criteria, including published data on reliability, validity, breadth of application, sensitivity to longitudinal change, and relevance to public health goal, as detailed and elaborated in Table 1. This rating uses criteria similar to those for almost all traditional health research measures. The second ‘Practical’ rating (which is a new addition to the traditional GEM and used only for D&I workspace measures at this time) considers the above criteria, but gives greater weight to pragmatic features related to the probability that the measure can be successfully used in real world settings such as primary care, state health departments, community projects, and low-resource settings, where there are many competing demands and limited research funds and/or staff to supervise data collection. Criteria for this second rating include feasibility, appropriateness, cost, and actionable results. These ratings and comments will be visible for each measure, and will be continuously updated on GEM and in other places to serve as a guide for the IS field in identifying what IS measures are appropriate for varying contexts. The intent is not to mandate the use of any specific subset of measures, but rather to inform selection by serving as a decision tool and provide a resource for those not familiar with measurement options to benefit from their colleagues’ experiences and knowledge.

**Table 1:** Proposed criteria for rating dissemination and implementation measures for scientific soundness and practicality

**GOLD STANDARD MEASURE RATING CRITERIA - For Primary Research Focus**	**PRACTICAL MEASURE RATING CRITERIA - For Real World Application**^**1**^
**Reliable**: Especially test-retest (less emphasis on internal consistency)	Feasible* Brief (generally 2 to 5 items or less); easy to administer/score/interpret
**Valid**: Construct validity, criterion validity, performed well in multiple studies	Important to Practitioners and Stakeholders* Relevant to health issues that are prevalent, costly, challenging; helpful for decision makers or practice
**Broadly Applicable:** Available in English and Spanish, validated in different cultures and contexts; norms available; no large literacy issues	**Actionable**: Based on information, realistic actions can be taken, *e.g.*, immediate discussion, referral to evidence-based on-line or community resources
Sensitive to Change* (if applicable) Longitudinal use, for performance tracking over time	**User Friendly:** Patient interpretability; face valid; meaningful to clinicians, public health officials, and policy makers
**Public Health Relevance:** Related to Healthy People 2020 goals, key IOM objectives or national priorities	Low Cost*: Publicly available or very low cost to use, administer, score, and interpret
	**Enhances Patient Engagement**: Having this information is likely to further patient engagement
	**Do No Harm:** Can likely be collected without interfering with relationships, putting respondents at risk, or creating unintended negative consequences

^1^ For use in pragmatic studies and real world settings where there are many competing demands, many other measures to assess For pragmatic rating, still consider gold standard criteria, but weight criteria on right most heavily.

NOTE: For both Gold Standard and Practical Measure scales, give criteria with *heaviest weighting in assigning ratings.

Finally, in phase 4, the celebrate phase, all the data collected during the campaign will be summarized and disseminated through hosted webinars, IS conferences, and newsletters to spark constructive conversation regarding harmonizing measures. Measures that are rated highly on average in either practicality or gold standard criteria may provide a basis for coming to consensus on potential key measures for the field, in conjunction with other rigorous evaluation methods. These conversations and resulting information can be used to provide guidance to D&I researchers and practitioners on which measures are most highly rated for different purposes and contexts, as well as present an opportunity to share the experiences of users who utilize different measures. At the time of this paper, the campaign was just entering phase 3 (this publication is part of the promotion for phase 3), so data on these later phases are not yet available.

### Evaluation

Google analytics and GEM usage reports were used to analyze progress and work done through the workspace’s associated measures and constructs. Metrics analyzed included: the cumulative number of measures and constructs in the GEM D&I Workspace; number of views [hits] and visitors for different sections; and measure information additions, edits, and downloads on a weekly basis (Additional file 1). Finally, a report was generated to assess the number, geographical location, and institutional affiliation of GEM registrants during this first period of the GEM D&I Campaign. These metrics, and the tools used, are typical for website evaluation and tracking.

### Results to date

By week 8 of the GEM D&I Campaign, there were a total of 45 constructs and 120 measures added to the GEM D&I Workspace. These measures are listed by construct in Additional file 3. The following constructs had the most associated measures: Organizational Culture (n = 13), Adoption (n = 8), and Acceptability (n = 7). There were 18 constructs that had only one measure including Reach, Organizational Change, and Fidelity. As of 14 May 2012, five constructs had no associated measures (*i.e.*, Demographics, Dissemination, Diffusion, Implementation Climate, Middle Managers Commitment To Innovation Implementation). There are, at present, 51 measures (43%) with uploaded instruments (full or partial). The lack of instrument upload was mostly due to a difficulty getting responses from the authors of proprietary measures to obtain the full instrument. Measures spanned a number of topic areas including process outcomes for D&I, characteristics of the innovation, evidence, policy, process, context, and target audience characteristics. Most of the uploaded measures targeted healthcare providers (including clinicians and other types of providers) (n = 37), the general population (including patients with different conditions; n = 27), or researchers (n = 18). Psychometric properties were reported for 58 measures (48%) with varying detail and completeness.

**Figure 4:**
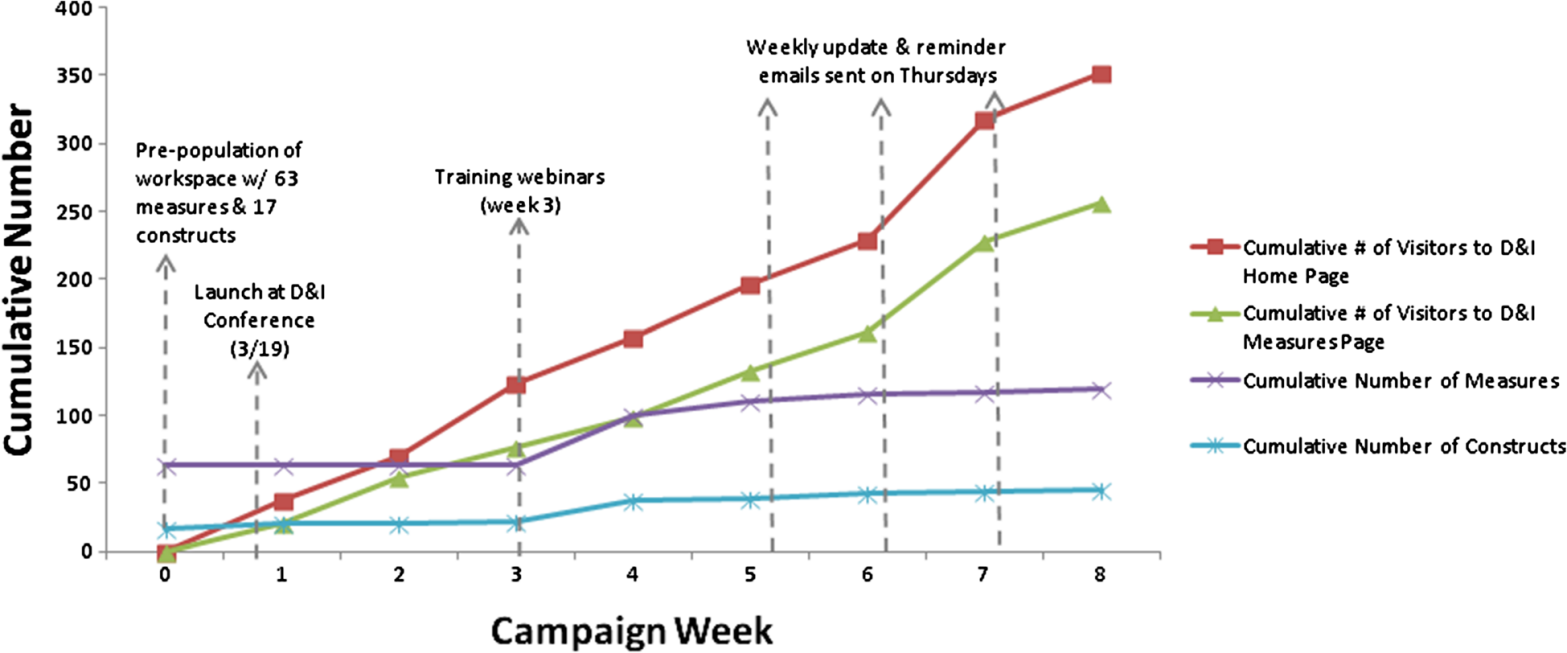
Cumulative results of the Grid-Enabled Measures Dissemination and Implementation Campaign by Campaign week.

The number of visitors on the GEM D&I Home Measures pages and the number of measures and constructs added to the GEM D&I Workspace were tracked on a weekly basis and are summarized as cumulative values in Figure 4. We observed peak activity periods for visits on week 7 (n = 89 visitors on GEM D&I Home page), and for number of added constructs and measures on week 4 (n = 16 for added constructs and n = 37 added measures). A total of 515 views from 351 visitors to the GEM D&I Workspace home page were counted during the campaign along with 501 views from 257 visitors to the GEM D&I Measures page.

The number of views, visitors, and downloads for each measure and construct page are provided in Additional file 3. A total of 4,721 views and 442 downloads were counted across all D&I measures. The most popular measures (based on their cumulative views and number of downloads) are summarized in Table 2. The top three measures based on views and downloads were the Morisky Eight-Item Medication Adherence Scale (n = 545 views, n = 128 downloads), Morisky Four-Item Self-Report Measure of Medication-Taking Behavior (n = 355 views, n = 96 downloads), Acceptability of Decision Aid Scale (n = 120 views), and Evidence Based Practice Attitude scale (n = 17 downloads).

**Table 2:** Most frequently accessed and downloaded Dissemination and Implementation (D&I) measures during the first 8 weeks of the Grid-Enabled Measures D&I Campaign

**Measure**	**# of Views**	**Measure**	**# of Downloads**
Morisky 8-Item Medication Adherence Scale	545	Morisky 8-Item Medication Adherence Scale	128
Morisky 4-Item Self-Report Measure of Medication-Taking Behavior (MMAS-4)	355	Morisky 4-Item Self-Report Measure of Medication- Taking Behavior (MMAS-4)	96
Acceptability of Decision Aid Scale	120	Evidence Based Practice Attitude scale	17
Evidence Based Practice Attitude scale	116	Perceived Competence Scale	16
Beliefs About Medicines Questionnaire	103	Atkinson's Perceived Attributes of eHealth Innovations	14
Absolute Concentration Index	102	Open Ended Goal Setting Tool	10
Atkinson's Perceived Attributes of eHealth Innovations	91	Chronic Illness Resources Survey (CIRS)	10
Perceived Competence Scale	82	RE-AIM Adoption measure	9
Care Transitions Measure	79	Goal Evaluation Tool (GET)	9
Stages of Implementation Completion measure	74	Health Literacy Screening Questions	9

A total of 109 individuals registered on GEM over the eight-week period of the GEM D&I Campaign. The majority of registrants (52.3%) were affiliated with academic institutions; the remainder came from cancer/medical centers (15.6%), private for profit and not-for-profit organizations (13.8%), and federal and state government (12.8%). The registrants predominantly came from the United States (US); however, 10% were international users and represented seven countries including Canada, the United Kingdom (UK), Malaysia, and Pakistan. Some participants in the GEM D&I Campaign had previously been registered members of GEM: 36 existing members logged in during the GEM D&I campaign. Due to limitations of the web analytics, we are unable to know whether these existing members or the newly registered members were associated with the GEM D&I campaign exclusively, because there was a second campaign related to Survivorship Care Planning that overlapped in time with the GEM D&I campaign. We counted 18 comments on the individual D&I measure and construct pages, and four comments on the GEM D&I discussion board. Most of these comments were about the usefulness and the psychometric properties of measures, the future use of these measures in research, and for whom the measures will be the most beneficial. Since the rating and commenting phase had not yet begun, we anticipate that the number of comments on the site will continue to increase over time.

The practicality rating dimension opens up a number of IS possibilities, and we plan to summarize the user feedback for both research and practitioner groups. A final opportunity that GEM has been developed to facilitate is sharing of actual datasets on D&I measures. If there is greater use of harmonized measures, this could provide a neutral, trusted platform for team science.

## Discussion

The key purposes of this paper were to describe a collaborative effort to enhance the quality and harmonization of measures for IS using a crowd sourcing approach through NCI’s GEM wiki-platform and to present results from the first eight weeks of the GEM D&I Campaign. As of 14 May 2012, the GEM D&I Campaign resulted in definitions for 45 constructs, summarized information on 120 measures, and generated 515 views from a total of 351 visitors on the GEM D&I Homepage. The spike in visitors observed during week 7 may have been due, in part, to a large international promotional push that occurred at that time with a Twitter posting, in key International D&I newsletters and list serves in the UK, Canada, and Australia. Furthermore, a total of 4,721 views and 442 downloads were counted across all measures.

The use of the existing GEM platform made it possible to initiate and implement the GEM D&I Campaign in a transparent, broad-reaching, systematic, rapid, and cost-effective fashion. The Campaign relied on collaboration across a not-for-profit research organization, an academic institution, and a government agency. The visibility of the Campaign was achieved using multiple channels and strategies, including the annual NIH/VA D&I conference, existing national and international networks of IS experts, use of Champions and opinion leaders, training, and ongoing technical assistance.

There were a number of challenges and lessons learned from the GEM D&I Campaign. Participation from outside of the US was modest (only 10 % of new registrants on GEM during the eight-week period were from countries outside of the US). Furthermore, most of the visitors to date were affiliated with academic institutions and cancer/medical centers, which indicates that engagement of population-based researchers has not been entirely successful. This was further confirmed by the nature of the most frequently visited and downloaded measures that had clear relevance to the medical context (*i.e.*, medication adherence). It is hoped that this publication, among other ongoing outreach activities, will address these issues.

While most of the added measures (110 out of 120 or 92%) are categorized according to the GEM criteria as ready for rating, there is a need for additional information on many measures, especially related to psychometric properties. Following submission of this manuscript, further work has been done to improve the availability of information on the site. Furthermore, mainly due to proprietary issues, only 43% of measures had actual instruments uploaded. Some IS constructs (*e.g.*, fidelity, dissemination) will also require the addition of example measures into GEM and will be the focus of future efforts. Measures that are not publically available to peers are less likely to become widely used and highly recommended.

Campaign engagement and usage analytics provide short-term feedback on the success of this collaborative effort, however, the long-term impact of the GEM D&I Campaign will not be measurable for another two to three years when use of harmonized IS measures within publications and grant applications might appear and can be tracked. Also, there are limitations to the exclusive reliance on automated usage data (Google analytics). Another limitation of the exclusive use of automated data is our inability to assess users’ satisfaction with and perceived usefulness of the GEM D&I Workspace. NCI has recently conducted usability testing of the GEM portal. The usability testing focused on having respondents navigate the site and provide feedback on its appearance and functionality and results will inform updates to the site.

While we observed a number of positive features associated with crowd sourcing, such as transparency, breadth of opinion and views, and iterative and participatory development, this campaign also evidenced some of the limitations of collaborative websites [18,19]. Most notably, while the GEM D&I Campaign has drawn a reasonable number of visitors over the first eight weeks of the campaign, most of the contributions to the GEM D&I Workspace were made by a smaller, core group of individuals. This is not an uncommon pattern in the use of Web 2.0 approaches [21] and is consistent with findings from other GEM workspace initiatives [16]. Web 2.0 research suggests the use of multi-faceted, tailored outreach efforts that combine individual and group-level outreach approaches to increase broader participation and encourage initial lurkers to gradually become more overtly engaged participants, contributors, and leaders [21].

A parallel, ongoing effort for the standardization and harmonization on measurement for IS should also be acknowledged. The Seattle Implementation Research Conference (SIRC) Instrument Review Project’s overall aim is to create a comprehensive repository of D&I measures and their instruments relevant to mental health research measuring the implementation outcomes identified by Proctor *et al.*[20] and to rate these measures using an expert panel approach focused on the degree of empirical validation (Personal communication with Cara C. Lewis, 13 March 2012.) The SIRC Instrument Review Project’s goals are similar to GEM’s, and while they differ in methods (*i.e.*, the use of a more systematic, expert approach versus a crowd sourcing collaborative participatory approach), both these initiatives are focused on the same goals—advancing the harmonization and rigor of measurement in IS. An advantage of GEM D&I is its integration within a larger effort around measure harmonization and its ‘home’ within the NIH-funded GEM website, giving it broader reach and visibility. Also, GEM encompasses a broader range of constructs, in that it spans all of NIH while SIRC is focused on mental health. Finally, SIRC is funded by an extramural grant, while GEM may have longer sustainability given its core NIH support and infrastructure. However, the GEM website (due to its wiki-based method) does not meet or seek to meet the criteria for a systematic, expert-based approach, including requiring complete information on all measures. We regard these two efforts as complementary rather than competitive and have started collaborative discussions with the SIRC group.

### Next steps and opportunities for engagement

As indicated in Figure 3, now that the GEM D&I Workspace has been sufficiently populated, the GEM D&I Campaign will focus on the rating and commenting (as well as providing added information on some of the incomplete measure characteristics). As the field of IS advances, and the number of participants engaged in GEM D&I grows, it makes sense to conceptualize the GEM D&I Campaign as an ongoing, circular, rather than a linear, effort with the potential of continuous expansion of the number of measures and constructs, and the addition of information on existing measures and constructs as they become available (see Figure 3). As such, knowledge can be gained and fed back to the community for further refinement.

Readers are invited to visit at http://www.gem-beta.org/GEM-DI and both rate and comment on measures areas across different IS constructs, as well as identify and add missing measures and constructs or complete metadata (*e.g.*, psychometric properties, items, response categories) on existing measures. Registration is required to promote transparency and accountability, and to encourage open discussion.

An innovation of the GEM D&I Campaign is the rating of measures using two scales: the Gold Standard (scientific soundness) rating scale and the Practicality rating scale (Table 1). The distinction of these two rating criteria is important as found in an earlier campaign on measures for routine use in electronic health records [16]http://conferences.thehillgroup.com/OBSSR/EHR2011/index.html and can be strengthened by engaging non-research stakeholders, including clinicians, policy makers, decision makers, and citizens, including patients and family members [22]. By participating in this effort, readers can not only share their own measures and benefit from measures uploaded by others, but can also receive recommendations and feedback from peers regarding measurement-related issues. The primary target audience for our efforts to date has been researchers with interest in IS and practice-based issues. Future efforts will include the involvement of non-academic stakeholders using our Clinical and Translation Science Award partners.

## Conclusions

There has been an increasing effort on synthesis and knowledge integration within the field of IS in the US as exemplified by resources like the now five-year-old annual NIH/VA D&I meeting (http://conferences.thehillgroup.com/obssr/di2012/about.html), a recently published authoritative book on D&I research in health edited by Brownson, Proctor, and Colditz [23], the monthly D&I e-newsletter produced by Wynne Norton (http://cancercontrol.cancer.gov/di_listserv/archive-2012-may.html), the multiple training and mentoring programs for D&I (TIDIRH, and the Research to Reality mentorship program --https://researchtoreality.cancer.gov/mentorship), IS (IRI; QUERI—http://www.queri.research.va.gov/ and CIPRS—http://www.queri.research.va.gov/ciprs/), and the publication of a recent manuscript on the role of such research supported by the NIH [24]. The purpose of the GEM D&I Campaign and this paper is to build on these emerging traditions and become an additional resource for the field of IS.

The GEM D&I Campaign has the potential to inform the field and provide directions for future funding by identifying gaps and specific content needs for future research and measures/methods development through funding agencies, such as the National Science Foundation, the NIH, and the Patient-centered Outcomes Research Institute, and to provide quality and performance measurement ideas and resources for accreditation, performance standards, and payment policy organizations, such as the Centers for Medicare and Medicaid Services, the National Committee for Quality Assurance, and the National Institute for Health and Clinical Excellence.

One promising approach to advance and expand the effectiveness of IS in the next decade is to rely on team science and crowd sourcing strategies, such as that used in the GEM D&I Campaign. The caveat of this paradigm is that it only works to the extent a large and informed group of participants contributes to the effort. We invite readers to become active participants and leaders of the GEM D&I community and contribute to the advancement of the IS field through the harmonization of definitions of constructs and use of standardized IS measures.

## Abbreviations

IS: Implementation Science; NCI: National Cancer Institute; GEM: Grid-Enabled Measures; D&I: Dissemination and Implementation; TMSP: Technology-Mediated Social Participation; DCCPS: Division of Cancer Control and Population Sciences; US: United States; UK: United Kingdom; NIH: National Institutes of Health; SIRC: Seattle Implementation Research Conference.

## Competing interests

The authors declare that they have no competing interests.

The opinions or assertions contained herein are the private ones of the authors and are not considered as official or reflecting the views of the National Institutes of Health.

## Authors’ contributions

**BAR** participated in the conceptualization and design of the GEM D&I Campaign, the pre-population of the GEM D&I, promotion of Campaign Phases, design of the analytic plan and data analysis and interpretation of data, co-led the writing of the manuscript, **PP** participated in the conceptualization and design of the GEM D&I Campaign, led the Campaign efforts, participated in the design of the analytic plan and data analysis and interpretation of data, led the writing of the *D&I Campaign Process* section and co-wrote other sections of the manuscript. **SN** participated the conceptualization and design of the GEM D&I Campaign, the promotion of Campaign Phases, design of the analytic plan and data analysis and interpretation of data, provided technical leadership for the creation of the GEM D&I Workspace, and co-led the writing of the *The Grid-Enabled Measures portal and the use of crowd sourcing/wiki approach to science* section and contributed to the writing of other sections of the manuscript. **RM** conceptualization and design of the GEM D&I Campaign, the promotion of Campaign Phases, design of the analytic plan and data analysis and interpretation of data, provided technical consultation for the creation of the GEM D&I Workspace, co-led the writing of the *The Grid-Enabled Measures portal and the use of crowd sourcing/wiki approach to science.* Section and contributed to the writing of other sections of the manuscript. **MH** participated in the design of the GEM D&I Campaign, the pre-population of the GEM D&I, the promotion of the Campaign Phases, participated in the preparation of the first draft of the manuscript and reviewed and provided feedback on the manuscript. **EKP** participated in the conceptualization and design of the GEM D&I Campaign, the pre-population of the GEM D&I, the promotion of the Campaign Phases, and reviewed and provided feedback on the manuscript. **RCB** participated in the conceptualization and design of the GEM D&I Campaign, the pre-population of the GEM D&I and reviewed and provided feedback on the manuscript. **REG** participated in the conceptualization and design of the GEM D&I Campaign, the pre-population of the GEM D&I, promotion of Campaign Phases, design of the analytic plan and data analysis and interpretation of data, and co-led the writing of the manuscript. All authors read and approved the final manuscript.

## Supplementary Material

Additional file 1 **Steps to Rate and Add Measures on GEM-D&I.** This file provides step-by-step instructions on how to both add and rate measures to the GEM-D&I workspace. Because the site had been updated slightly between initial launch of the workspace and publication, this is a updated version of the instructions sent to the interested users as part of the campaign toolkit and used in trainings and webinars. Click here for file

Additional file 2 **D&I GEM Factsheet.** This file provides an overview of the Dissemination and Implementation Measures and Methods Initiative and was used for promotional purposes throughout the D&I GEM campaign, including as a component of the campaign toolkit sent to interested users. Click here for file

Additional file 3 **Dissemination and Implementation (D&I) Measures on the Grid-Enabled Measures Wiki Platform and affiliated analytics.** This file summarizes all constructs and affiliated measures and related analytics including hits, visitors, editing hits, comments, and downloads that were entered into the GEM D&I Workspace by 14, May 2012. Click here for file
