# Supplementary material for: Advancing the application, quality and harmonization of implementation science measures
Source: Implement Sci. 2012 Dec 11;7:119. doi: 10.1186/1748-5908-7-119 (PMC3541131; doi:10.1186/1748-5908-7-119)
Supplement: Additional file 1 — Steps to Rate and Add Measures on GEM-D&I. This file provides step-by-step instructions on how to both add and rate measures to the GEM-D&I workspace. Because the site had been updated slightly between initial launch of the workspace and publication, this is a updated version of the instructions sent to the interested users as part of the campaign toolkit and used in trainings and webinars. [file 1748-5908-7-119-S1.pdf]

IMPLEMENTATION SCIENCE
INTEGRATING SCIENCE, PRACTICE, AND POLICY

GEM
Grid-Enabled
Measures Database

The Dissemination and Implementation Measures Initiative has had approximately 125 measures across 40 constructs added to the site. Now we want to shift our focus to rating and commenting on those measures. The feedback we are asking you to provide is both quantitative and qualitative, as you will rate measures on two criteria (see table below) and can provide comments to elaborate on your rating. For more instruction watch a brief video: http://www.youtube.com/watch?v=LrbsB7KA-nQ

## How do I Rate/Comment on a Measure? (screen shots on page 2)

1) Go to the GEM-DI Workspace (https://www.gem-beta.org/GEM-DI) and **log in**.
    a. If you are a first-time user, click the box in the top left-hand corner to "Register".
    b. If already a member, but you forgot you password, click "Forgot Password" on log-in screen.
2) Click on the "Measures" tab on the left-hand side of the screen (or use the attached Excel file to link directly to one)
    a. Select the construct/measure that you want to assess. You are encouraged to rate as many measures as you possible within a construct (e.g. Implementation) for comparative purposes.
    b. By clicking through the tabs on the left hand of the screen, you will also be able to view details about other measure characteristics, such as its psychometric properties, reliability, and validity as well as history, language translations, etc. Additionally, each measure will have references to provide more information.
    c. If an instrument for a measure is publicly available, a PDF icon will appear on the right hand of the screen, indicating that it is available for download to review prior to rating.
3) When you are ready to assign your rating, click on the stars by "Avg. Rating" at the top-right of the page to rate or click on the left tab "Comments/Ratings". You will be taken to the tab to enter your rating/comments.
4) For the "**Gold Standard**" **criteria** (reliability, validity, etc), use the available radio buttons to rate the measure on the 5-point scale (1 being lowest, five being highest). See the left column in table below for criteria to take into consideration when assigning a rating score for "Gold Standard".
5) For the "**Practical Measure" criteria**, there are no radio buttons. Instead, in the open comments box, assign a numerical score between 1 and 5 (1 being low practicality, five being high practicality). See right column in table below for criteria.
6) You can also enter any additional qualitative comments in the text box to explain both your Practical and Gold Standard scores or ask questions of the community.
7) When you are all done, **be sure to click "Save"** to submit your rating and comments

| "GOLD STANDARD" MEASURE RATING CRITERIA - For Primary Research Focus | PRACTICAL MEASURE RATING CRITERIA - For Real World Application [1] |
|---|---|
| **Reliable**: Especially test-retest (less emphasis on internal consistency) | **Feasible*** Brief (generally 2-5 items or less); easy to administer/score/interpret |
| **Valid**: Construct validity, criterion validity, performed well in multiple studies | **Important to Practitioners and Stakeholders*** Relevant to health issues that are prevalent, costly, challenging; helpful for decision makers or practice |
| **Broadly Applicable:** Available in English and Spanish, validated in different cultures and contexts; norms available; no large literacy issues | **Actionable**: Based on information, realistic actions can be taken, e.g., immediate discussion, treatment/counseling or referral to evidence-based on-line or community resources |
| **Sensitive to Change*** (if applicable) Longitudinal use, for performance tracking over time | **User Friendly:** Patient interpretability; face valid; meaningful to clinicians, public health officials, and policy makers |
| **Public Health Relevance:** Related to Healthy People 2020 goals, key IOM objectives or national priorities | **Low Cost*:** Publicly available or very low cost to use, administer, score, and interpret |
| | **Enhances Patient Engagement**: Having this information is likely to further patient engagement |
| | **Do No Harm:** Can likely be collected without interfering with relationships, putting respondents at risk, or creating unintended negative consequences |

**NOTE**: For both "Gold Standard" and Practical Measure Use, give criteria with * heaviest weighting in assigning ratings
[1] For use in pragmatic studies and real world settings where there are many competing demands, many other measures to assess etc.

## Measures Detail Page:

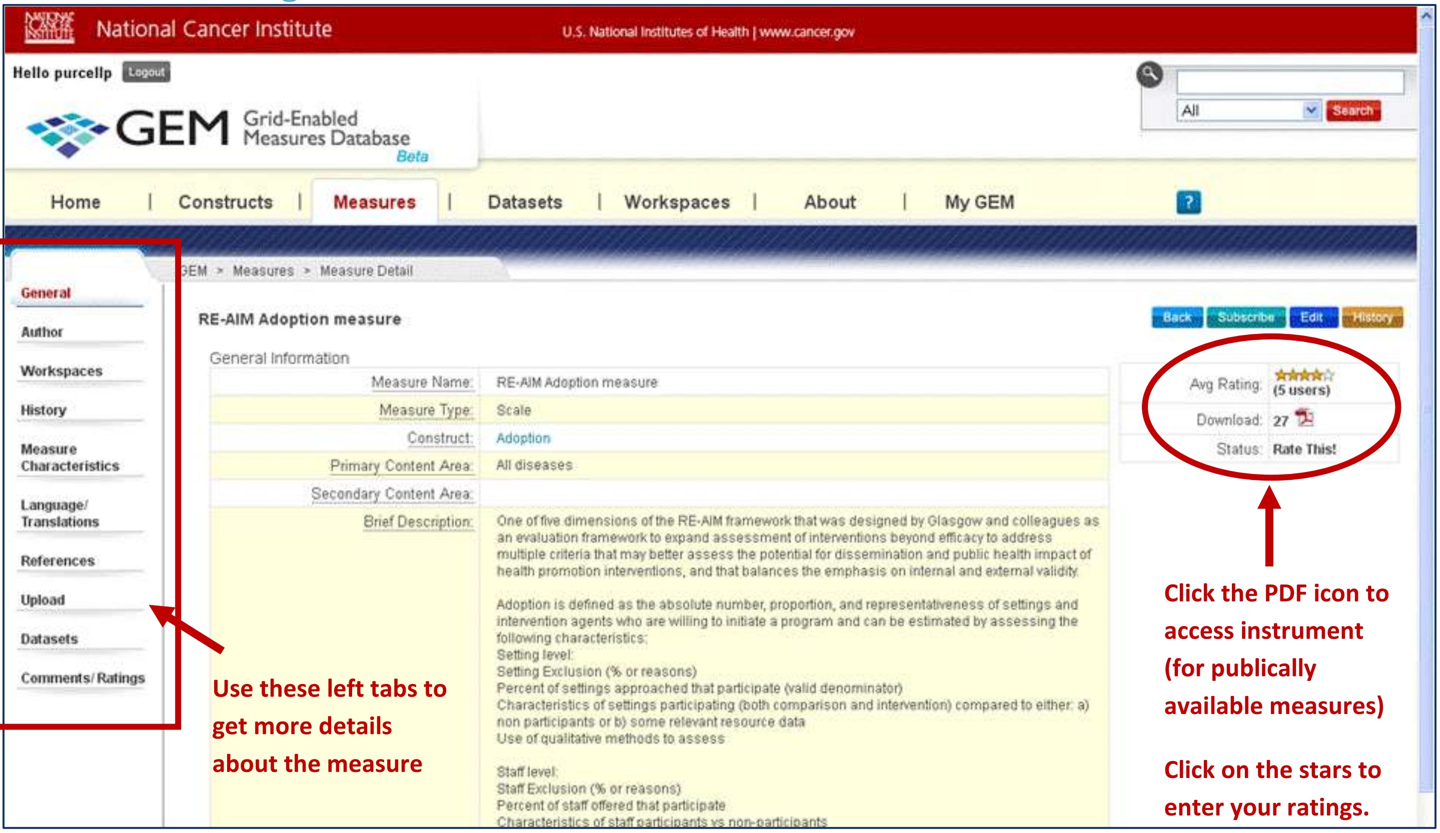


## Ratings/Comments Page:

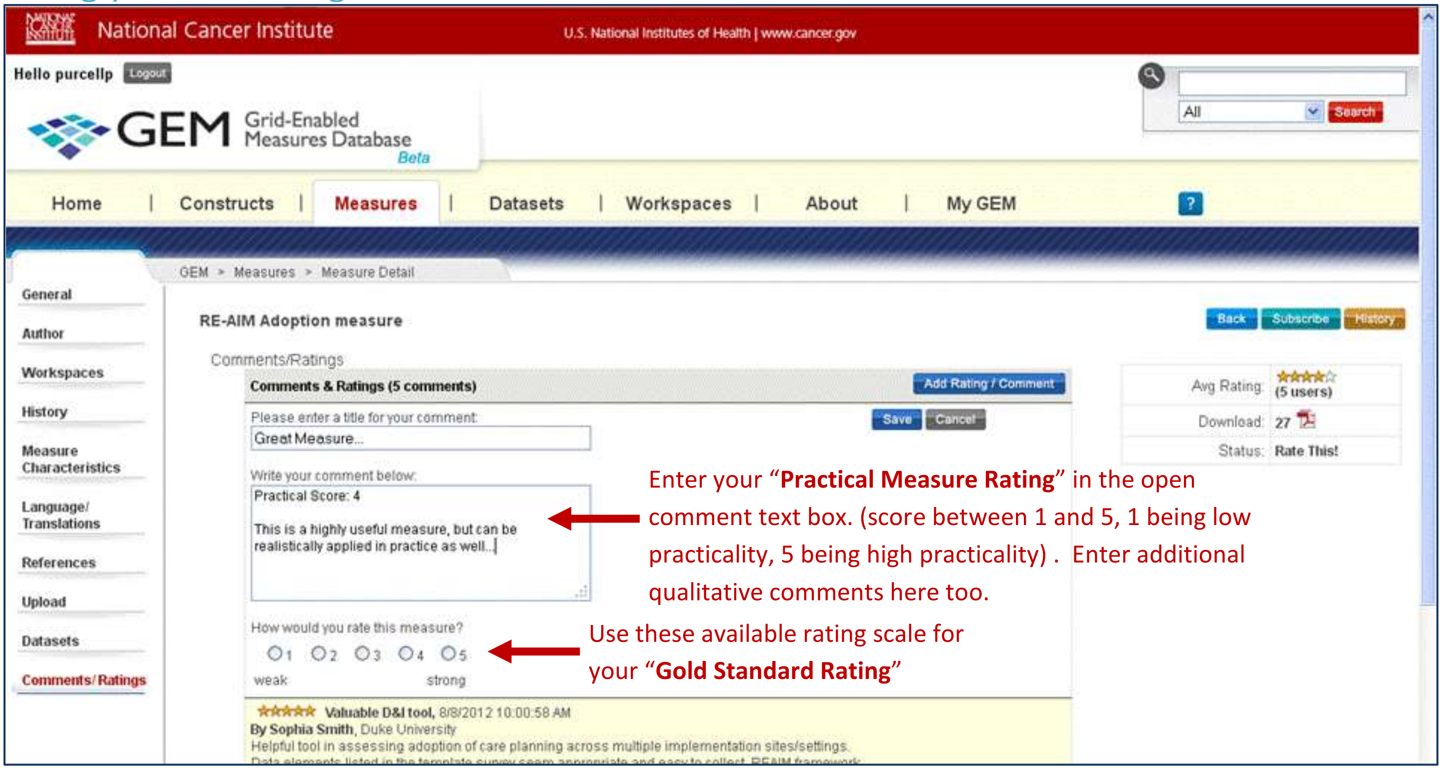

## Don't see a measure you think should be in the database? Here's how you can add a measure:

1) Visit the GEM website at www.gem-beta.org and log in with your username. If you are a first-time user, click the box in the top left-hand corner to "Register." Forgot your password? Just click on the "Forgot Your Password" hyperlink on the log-in screen.

2) Once logged in, go to the Measures tab and click the "Add New Measure" button. (**Note:** You may want to do a search *(upper right hand of GEM site)* for the name of the measure first to make sure it doesn't already exist in the database)

3) Enter as much information as possible about the measure, clicking the "Save and Continue" button at the bottom of each page, and using the left tabs to go to the next section.
   - Tabs include: General Information, Author Information, Workspace, References and Publications, and Upload Measure, which allows you to upload a publicly available measure as a pdf file so others can download it.

4) To link a new measure, or one already in the overall GEM database, to the GEM-D&I Workspace, select "The GEM-Dissemination and Implementation Initiative (GEM-DI)" from the drop-down menu in the Workspaces section of measures detail page (3rd tab), and click on the "Add Workspace" button on the right.

5) **IMPORTANT:** Click "Review and Submit" when you are finished entering information about a measure, or "Back" to make changes to the information you entered..

**NOTE:** The minimum requirements needed to add a measure to GEM are: 1) Measure Name, 2) Construct (you may need to add an additional construct if the appropriate one isn't listed), and 3) Corresponding Author(s); however, it is highly encouraged to add as much information as you can and to upload the actual measure or instrument for others to view.

You can also join a discussion started by Dr. Russ Glasgow about D&I methods and context on Research to Reality (R2R).

Visit: http://cancercontrol.cancer.gov/IS/dimmi.html for more information about this initiative.

If you have any questions, please contact **Peyton Purcell at purcellp@mail.nih.gov**.

**Please help us advance the field by adding and rating measures today! The success of the D&I Initiative depends on you.**

## Initiative Sponsors:

Cancer Communication Research Center

Centers of Excellence in Cancer Communication Research CECCR

NATIONAL CANCER INSTITUTE
