# Supplementary material for: Advancing the application, quality and harmonization of implementation science measures
Source: Implement Sci. 2012 Dec 11;7:119. doi: 10.1186/1748-5908-7-119 (PMC3541131; doi:10.1186/1748-5908-7-119)
Supplement: Additional file 2 — D&I GEM Factsheet. This file provides an overview of the Dissemination and Implementation Measures and Methods Initiative and was used for promotional purposes throughout the D&I GEM campaign, including as a component of the campaign toolkit sent to interested users. [file 1748-5908-7-119-S2.pdf]

National Cancer Institute

Implementation Science
Integrating Science, Practice, and Policy

What is the D&I Measures and Methods Initiative?

The purpose of the Dissemination and Implementation (D&I) Measures and Methods initiative is to bring together an international community of researchers and practitioners to create a growing and evolving resource for standardized, vetted D&I measures that can lead to comparable datasets and facilitate collaboration and comparison across disciplines and regions.

The D&I Measures and Methods Initiative and resource enables researchers and practitioners to:

- Identify and define constructs relevant to D&I research and practice;
- Learn about, comment on, and rate existing measures for D&I;
- Share new D&I measures;
- Identify missing D&I measures;
- Learn about strategies/methods relevant to D&I

Initiative Components:

- D&I Grid-Enabled Measures (GEM) Workspace – interactive resources for D&I constructs and measures.
- D&I Methods Table– listing of data collection, analysis, and design methods relevant to the D&I field.

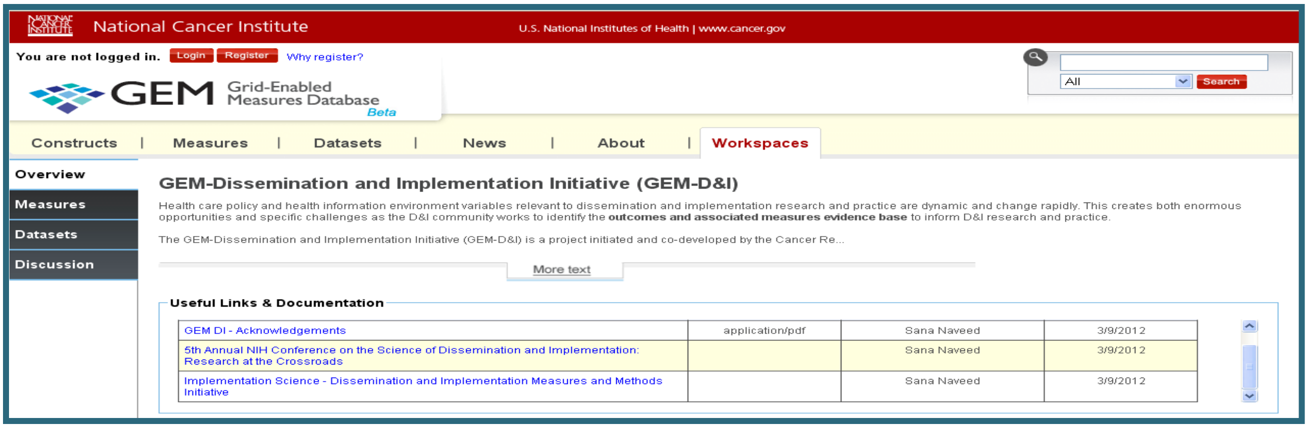

Why should I get involved?

If you are interested in advancing the D&I field, this Initiative is an excellent way to contribute to the field and engage with colleagues. The D&I Measures and Methods Initiative gives you access to D&I constructs, measures, and methods developed by other colleagues and also provides you with a platform to share your own D&I measures.

How can I get involved?

- Visit the D&I GEM Workspace (www.gem-beta.org/GEM-DI), click on "Workspaces" to review, comment on, and rate existing D&I measures and constructs, and add additional ones.
- Review the D&I methods tables (http://cancercontrol.cancer.gov/IS/dimmi.html) and join the discussion on the Research to Reality (researchtoreality.cancer.gov) community of practice on these approaches to provide feedback and share your experiences.

Join the initiative:

http://cancercontrol.cancer.gov/IS/dimmi.html or www.gem-beta.org/GEM-DI

U.S. DEPARTMENT OF HEALTH AND HUMAN SERVICES

National Institutes of Health

Cancer Communication Research Center

Centers of Excellence in Cancer Communication Research CECCR

National Cancer Institute

Use the QR code above to learn more
