# Supplementary material for: Advancing the application, quality and harmonization of implementation science measures
Source: Implement Sci. 2012 Dec 11;7:119. doi: 10.1186/1748-5908-7-119 (PMC3541131; doi:10.1186/1748-5908-7-119)
Supplement: Additional file 3 — Dissemination and Implementation (D&I) Measures on the Grid-Enabled Measures Wiki Platform and affiliated analytics. This file summarizes all constructs and affiliated measures and related analytics including hits, visitors, editing hits, comments, and downloads that were entered into the GEM D&I Workspace by 14, May 2012. [file 1748-5908-7-119-S3.pdf]

Additional file 1 - Dissemination and Implementation (D&I) Measures on GEM Wiki Platform and affiliated analytics (data as of 5/14/2012)

Construct	Measures of Construct	# of Hits	# Visitors	# of editing hits	# of Comments	# of Downloads
Acceptability		144	109	8	1	N/A
	Van Schaik's Technology acceptance	37	23	15	--	5
	Evidence Based Practice Attitude scale	116	78	24	--	17
	Suicide Prevention Program Rating Profile	11	6	7	--	--
	Therapists' attitudes toward treatment manuals	27	18	12	--	3
	Treatment Evaluation Inventory	70	51	22	--	5
	Residents incorporation of EBM in practice	19	14	10	--	2
	Atkinson's Perceived Attributes of eHealth Innovations	91	51	19	1	14
Adherence		233	115	99	--	N/A
	Questionnaire on the Quality of Physician-Patient Interaction	23	18	9	--	--
	Medical Outcomes Study (MOS) Measures of Adherence	24	16	5	--	--
	Morisky 8-Item Medication Adherence Scale	545	372	41	--	128
	Morisky 4-Item Self-Report Measure of Medication-Taking Behavior (MMAS-4)	355	199	44	--	96
Adopter Attitudes		14	12	1	--	N/A
	Steckler's Perception of Innovation "Roger's Adoption Questionnaire	39	24	17	--	3
Adoption		58	36	92	--	N/A
	Roman's measure of adoption in substance abuse treatment	17	11	11	--	1
	Haug's Measure of Evidence-Based Practice Adoption	33	27	10	--	--
	Nursing Practice Questionnaire	11	9	7	--	--
	Li, Simon, Bodenheimer, Gillies, Casalino, Schmittdiel, & Shortell, adoption	19	12	8	--	1

	Menachemi, Saunders, Chukmaitov, Matthews, & Brooks	16	11	10	--	1
	Noonan, Emshoff, Mooss, Armstrong, Weinberg, & Ball, Adoption, Adaptation, and Fidelity	22	12	11	--	2
	Zaidi, Marriott, & Nation, adoption, acceptability and appropriateness	56	20	16	--	--
	RE-AIM Adoption measure	70	42	27	--	9
Advancing Women Scientists		3	3	20	--	N/A
	ADVANCE Scale	36	18	20	--	--
Appropriateness		41	25	39	--	N/A
	Nancy Atkinson scale of perceived attributes of eHealth innovations	24	17	9	--	1
	Workshop Evaluation	29	19	10	--	6
	Zaidi's survey of web-based antibiotic approval systems	12	7	5	--	1
	Pankratz perceived attributes of a federal drug prevention policy scale	13	9	10	--	1
	Cognitive behavior and Monitoring Scale	15	10	5	--	2
Care Coordination		73	61	19	1	N/A
	Care Transitions Measure	79	61	12	--	--
	Usual Provider Continuity Index	33	24	52	1	--
	Continuity of Care Index	27	19	30	--	--
	Perception of Continuity (PC) Scale	41	32	38	--	--
Chronic Illness Care		35	35	5	--	N/A
	Patient Assessment of Chronic Illness Care (PACIC)	51	33	12	--	3
Collaborative Service Delivery		10	10	6	--	N/A
	Assessing Readiness for Collaborative Service Delivery	36	23	6	--	--
Comorbidity		24	19	3	--	N/A
	Questionnaire to Verify Stroke Free Status	8	5	18	--	--
Continuous Quality Improvement		18	15	3	--	N/A
	Organizational-Level Processes of Change Scale	35	27	10	--	--

Cost		18	14	21	--	N/A
	Recruitment Costs	13	12	3	--	--
	Intervention Costs	21	14	6	--	--
	Implementation Costs	41	32	5	--	--
	Cost-effectiveness Ratio	38	31	7	--	--
Demographics		31	21	7	--	--
Diffusion		22	18	9	--	--
Dissemination		40	32	18	--	--
Evidence-based Quality Improvement		3	3	2	--	N/A
	ACE EBP Readiness Inventory	27	19	40	--	--
Feasibility		32	23	4	--	N/A
	Vandelanotte & De Bourdeaudhuij, Feasibility & Acceptability	37	29	10	--	4
	Nemeroff, Feasibility	19	16	9	--	1
	Martens, Feasibility & Acceptability	21	17	6	--	2
	Ondersma, Chase, Svikis, Schuster, Feasibility & Acceptability	32	26	10	--	1
Fidelity		18	18	--	--	--
	TMACT - Tool for Measurement of Assertive Community Treatment	--	--	--	--	--
Health Disparities		26	15	9	--	N/A
	Index of Disparity by Pearcy and Keppel	57	24	48	--	--
	Range Ratio	43	14	82	--	--
	Range Difference	40	10	71	--	--
	Theil Index	40	16	50	--	--
	Absolute Concentration Index	102	38	79	--	--
	Mean Log Deviation	31	11	42	--	--
	Between-Group Variance	33	11	46	--	--

	Health Disparities Calculator	21	14	34	--	--
Health Literacy/Numeracy		43	33	4	--	N/A
	Health Literacy Assessment Using Talking Touchscreen Technology (Health LiTT)	30	13	4	--	--
	Health Literacy Skills Instrument	30	20	4	--	--
	Health Literacy Screening Questions	36	22	5	--	9
	Patient Literacy Preferences	16	11	5	--	3
	Health Literacy Skills Instrument Short Form	27	20	7	--	--
	Subjective Health Literacy and Numeracy	24	15	5	--	7
Illness Representations		9	8	35	1	N/A
	Beliefs About Medicines Questionnaire	103	78	35	1	--
Implementation		48	39	10	1	N/A
	Stages of Implementation Completion measure	74	45	51	--	5
	Promoting Action on Research Implementation in Health Services (PARiHS)	51	32	15	--	--
	PPC-RS	8	6	49	--	--
	RE-AIM Implementation measure	42	29	16	--	--
Implementation Climate		25	17	12	--	--
Maintenance		8	7	11	--	N/A
	RE-AIM Maintenance Measure	20	11	11	--	6
‘Middle managers' commitment to innovation implementation		11	9	6	--	--
Organizational and Group Communication		14	13	4	--	N/A
	Group Development Questionnaire	17	8	14	--	--
Organizational Capacity		33	27	63	--	N/A
	Is Research Working for You?	30	22	3	--	--
	McKinsey Capacity Assessment Grid	52	24	60	--	5

Organizational Change		26	19	11	--	N/A
	NCDDR's Dissemination Self-Inventory	6	5	39	--	--
Organizational Climate		41	26	14	--	N/A
	Glisson's Organizational Social Context (OSC)	58	43	3	--	--
	Steckler's Organizational Climate	25	16	17	--	--
Organizational Culture		63	40	23	--	N/A
	Practice Culture Questionnaire	35	26	2	--	--
	MacKenzie's Culture Questionnaire	22	19	2	--	--
	Core Employee Questionnaire	21	15	2	--	--
	Competing Values Framework	44	30	2	--	--
	Quality Improvement Implementation Survey	21	16	6	--	1
	Organizational Culture Inventory	38	29	52	--	--
	Harrison's Organizational Ideology Questionnaire	47	38	5	--	--
	Hospital Culture Questionnaire	10	7	12	--	--
	Nursing Unit Cultural Assessment Tool	7	6	2	--	--
	Survey of Organizational Culture	24	20	3	--	--
	Corporate Culture Questionnaire	22	13	3	--	--
	Hofstede's Organizational Culture Questionnaire	47	42	8	--	--
	Organizational Culture Survey	29	23	12	--	--
Organizational Readiness for Change		33	27	2	--	N/A
	Organizational Readiness-to-Change Assessment (ORCA)	61	41	39	--	7
	CPCQ (Change Process Capability Questionnaire)	17	13	46	--	--
Partnership		12	10	10	--	N/A
	PARTNER (Program to Analyze, Record, and Track Networks to Enhance Relationships)	13	9	30	--	--
	Partnership Self-Assessment Tool	9	8	25	--	--
	Human Services integration measure	8	7	20	--	--
	Review Criteria and Rating Scale for Community-Based	8	6	21	--	1

	Participatory Research					
Patient Goals		39	28	4	--	N/A
	Open Ended Goal Setting Tool	40	21	9	--	10
	Patient's Preferred Method for Communication	23	16	9	--	1
	Goal Evaluation Tool (GET)	37	22	8	--	9
Patient Satisfaction with Decision Aid		27	22	33	1	N/A
	Acceptability of Decision Aid Scale	120	76	33	1	--
Penetration		13	11	4	--	N/A
	Langabeer's Questionnaire	11	7	2	--	--
	Woltmann's Index of Penetration	12	9	2	--	--
	Shediac-Rizkallah's Measure of Penetration	7	5	2	--	--
	Bartholomeu Workshop Evaluation and Workshop Assessment Follow-Up	11	9	7	--	--
	Stile's Measure of Penetration	6	5	2	--	--
Perceived Autonomy Support		5	5	16	1	N/A
	Perceived Autonomy Support	43	27	16	1	4
Perceived Competence Scale		20	19	18	--	N/A
	Perceived Competence Scale	82	48	18	--	16
Pragmatism		2	2	4	--	N/A
	PRECIS (Pragmatic-Exploratory Continuum Indicator Summary)	10	6	40	--	--
Quality of Life		73	61	11	1	N/A
	Veterans RAND 12 Item Health Survey (VR-12)	33	28	8	--	--
	EQ-5D	15	10	3	--	5
	Behavioral Risk Factor Surveillance System- Quality of Life	21	14	5	--	5
	SF-12	10	8	6	--	--
	PROMIS Global Physical Health (GPH) Short Survey	34	18	22	1	6
Reach		6	5	4	--	N/A

	RE-AIM Reach Measure	26	16	11	--	3
Research Utilization		15	11	53	--	N/A
	Level of Research Utilization in Policy-Making	27	17	12	--	4
	Hall's Levels of Use Scale	34	23	13	--	2
	Landry's Use of Social Science Research	40	15	20	--	--
	Landry's Knowledge Utilization Scale among Policymakers	27	12	11	--	4
Social Support		32	30	4	--	N/A
	The Social Support Questionnaire	37	28	5	--	--
	Chronic Illness Resources Survey (CIRS)	34	21	7	--	10
	ENRICHD Social Support Inventory (ESSI)	34	27	11	--	--
	Medical Outcomes Study-Social Support Survey	11	8	4	--	--
	Wisconsin Social Support Scale	22	20	6	--	--
Sustainability		22	16	1	2	N/A
	Program Sustainability Assessment Tool	30	25	6	--	--
	Goodman's Level of Institutionalization	56	28	20	2	4
Treatment Self-Regulation		18	14	25	--	N/A
	Treatment Self-Regulation Questionnaire	35	20	25	--	5
Adapted from https://www.gem-beta.org/public/wsmeasures.aspx?cat=8&aid=1&wid=11						
